# Overview of the consortium of hospitals advancing research on tobacco (chart)

**DOI:** 10.1186/1745-6215-13-122

**Published:** 2012-08-01

**Authors:** William T Riley, Victor J Stevens, Shu-Hong Zhu, Glen Morgan, Debra Grossman

**Affiliations:** 1 National Heart, Lung, and Blood Institute, 6701 Rockledge Drive, MSC 7936, Bethesda, MD, 20892, USA; 2 Kaiser Permanente Center for Health Research, 3800 N. Interstate Ave, Portland, OR, USA; 3 University of California, San Diego, San Diego, CA, USA; 4 National Cancer Institute, 6130 Executive Blvd, Bethesda, MD, USA; 5 National Institute on Drug Abuse, 6001 Executive Blvd, Bethesda, MD, USA

**Keywords:** Hospitals, Smoking cessation, Tobacco control

## Abstract

**Background:**

The Consortium of Hospitals Advancing Research on Tobacco (CHART) is a network of six projects and a research coordinating unit funded by the National Heart, Lung, and Blood Institute, the National Cancer Institute, the National Institute on Drug Abuse, and the National Institutes of Health (NIH) Office of Behavioral and Social Science Research. The CHART projects will assess the effectiveness and cost-effectiveness of smoking cessation interventions initiated during hospitalization and continued post-discharge.

**Methods/design:**

Along with a seventh project funded previously under the NIH Challenge grants, the CHART projects will assess smoking cessation strategies delivered to approximately 10,000 hospitalized smokers across a geographically diverse group of nearly 20 private, public, academic, and community hospitals. The CHART research coordinating unit at Kaiser Permanente Center for Health Research provides organizational and data coordination support, facilitating the development of common measures for combining data from multiple CHART projects.

**Discussion:**

The targeted enrollment in CHART, if achieved, will represent the largest, most diverse pooled dataset of hospitalized smokers receiving smoking cessation assistance, and is designed to contribute to the dissemination and implementation of smoking cessation interventions provided by hospital systems.

## Background

Hospitalization provides numerous advantages as a setting for smoking cessation interventions. Many hospitalized smokers are admitted due to a smoking-related illness, providing a serious and salient motivational prompt for quitting smoking. During hospitalization, patients have access to a range of health care staff potentially able to provide various smoking cessation services. Hospitalization also provides a compulsory period of abstinence, particularly in hospitals with well enforced smoke-free campus policies, that provides patients with a “head start” in the quitting process.

By leveraging these advantages, a number of studies have shown that behavioral and pharmacologic smoking cessation interventions provided to hospitalized smokers are efficacious in improving cessation rates. In a recent meta-analysis Rigotti and colleagues [1] found that smoking cessation interventions initiated during hospitalization and continued post-discharge for at least 1 month increase the odds of long-term cessation by 65%. A recent meta-analysis with Monte Carlo modeling of cost-effectiveness estimated that smoking cessation counseling with follow-up contact for all US smokers hospitalized with acute myocardial infarction (AMI) would cost $540 per quitter to implement and would prevent 1,380 nonfatal AMIs and 7,860 deaths [2]. Given the clear positive impacts of smoking cessation services provided during and following hospitalization, the Joint Commission recently updated its performance measures to include inpatient and post-discharge provision of smoking cessation counseling and medications to all patients effective 1 January 2012 [3].

The National Heart, Lung, and Blood Institute (NHLBI) convened a workgroup in 2008 to evaluate the state of the science in this area and to consider directions for future research. They considered various types of smoking cessation services for hospitalized patients and the barriers to their implementation [4]. This workgroup noted the need for effectiveness and cost-effectiveness trials using broader, hospital-wide interventions that could be easily adopted and integrated into hospital systems. Based on these workgroup findings, the NHLBI, along with the National Cancer Institute (NCI), National Institute on Drug Abuse (NIDA) and the National Institutes of Health (NIH) Office of Behavioral and Social Science Research (OBSSR), released a request for applications (RFA) on “Effectiveness Research on Smoking Cessation in Hospitalized Patients (U01)” (RFA-HL-10-020 and RFA-HL-10-025). The purpose was to encourage research to evaluate the translation of efficacious smoking cessation strategies initiated during hospitalization and continued post-discharge into effective programs that can be widely implemented in routine clinical practice, and assess the cost-effectiveness of these interventions.

## Methods/design

### Overview of the CHART collaborative group

From the RFAs, six cooperative agreements and a research coordinating unit (RCU) were funded. In addition, a previously funded NIH Challenge grant (RC1 HL 099668) was invited to join the CHART network. Table 1 lists the grantee institutions, the hospitals participating in the consortium, and a brief description of the interventions being compared to usual care in these seven projects. The CHART projects include hospitals from every region of the US, representing academic medical centers, community public hospitals, and private hospitals. Combined enrollment in the CHART studies is expected to include approximately 10,000 hospitalized smokers.

**Table 1:** Consortium of Hospitals Advancing Research on Tobacco (CHART) projects

**Grant**	**PI**	**Institution**	**Hospitals (number)**	**Total N**	**intervention description**
U01 DA 031515	Bailey	University of Alabama Birmingham	UAB University Hospital (one)	1488	Post-discharge interactive web-based program that offers tailored information, e-group support, and text messages
U01 HL 105218	Duffy	University of Michigan	Trinity Health System Community Hospitals (six)	2350	Nurse-administered Tobacco Tactics intervention during hospitalization with post-discharge follow-up via trained volunteers
U01 HL 105231	Fellows	Kaiser Foundation Research Institute	Kaiser Portland, Oregon Health Sciences University Hospital (two)	900	Post-discharge IVR-supported assisted referral integrated with the hospital information system.
U01 HL 105232	Richter	University of Kansas Medical Center	University of Kansas Medical Center Hospital (one)	994	Warm handoff – Assist patient in making initial quitline contact while in hospital
RC1 HL 099668	Rigotti	Massachusetts General Hospital	Massachusetts General Hospital (one)	330	Post-discharge IVR assisted telephone counseling x 3 mos. plus a 30 day supply of smoking cessation medication
U01 HL 105229	Sherman	New York University	Bellevue, Manhattan VA (two)	3100	Post-discharge multi-session telephone counseling by hospital smoking cessation staff
U01 CA 159533	Zhu	University of California San Diego	Scripps Mercy, Chula Vista, and Green Hospitals, and UCSD Hillcrest and La Jolla hospital (five)	1600	2 x 2 factorial design comparing post-discharge NRT, proactive telephone counseling, and combined

IVR, interactive voice response; NRT, nicotine replacement therapy; PI, Principal Investigator.

The interventions evaluated by these projects are diverse. While some intervene primarily during hospitalization (for example, Duffy and Richter), others intervene primarily during post-discharge follow-up (for example, Zhu and Bailey). Post-discharge cessation counseling is provided via conventional telephone counseling (for example, Zhu, Sherman and Richter) as well as by more innovative methods such as internet websites (for example, Bailey) and interactive voice response (for example, Fellows and Rigotti). Some provide cessation medications for a specified period of time post-discharge to all for whom they are indicated while other interventions do not provide cessation medications but may include efforts to encourage attending physicians to include these medications in their discharge orders. Most of the projects are standard two-arm randomized clinical trials comparing active intervention to usual care; however, the Zhu protocol uses a factorial design to test the effects of nicotine replacement and telephone counseling alone and in combination, and the Duffy protocol randomly assigns hospitals to receive nurse training in smoking cessation interventions.

### Research coordinating unit and consortium governance structure

Although each project tests different interventions in different hospital settings, the RFA specified a cooperative agreement in which pooled data across studies could be used to address questions beyond the scope of any individual project. To facilitate project coordination, a companion RFA solicited for a RCU to organize and support network functions, including planning meetings, supporting communication and document sharing throughout the consortium, supporting communications with the consortium’s Data and Safety Monitoring Board (DSMB), and facilitating the development and implementation of a common set of baseline and follow-up measures to be used by the CHART projects. The RCU was awarded to Kaiser Permanente Center for Health Research in Portland, Oregon (U01 HL 105233, Principal Investigator (PI ) Victor Stevens).

The CHART organizational structure, which was specified by the RFA, closely matched the structure used in the Obesity Prevention at the Worksite consortium [5]. The RCU PI currently serves as chair of the CHART Steering Committee (SC). The voting members of the SC include the PIs of each research project and the lead project scientists of the three primary funding institutions (NHLBI, NCI, and NIDA). The SC meetings are open to other project investigators and staff. The SC meets monthly by conference call and at least twice a year in person. The SC sets all policies for the consortium, creates sub-committees as needed, and is responsible for resolving any issues that may arise within the consortium.

The CHART SC created the following subcommittees: 1) *Design and Analysis subcommittee* responsible initially for considering and recommending common inclusion/exclusion criteria and common baseline and outcome measures across projects; 2) *Cost-Effectiveness subcommittee* within the Design and Analysis subcommittee responsible for considering and recommending common cost-effectiveness data collection procedures across projects; 3) *Recruitment and Retention subcommittee* responsible initially for determining and recommending common procedures for monitoring and reporting of the recruitment and enrollment process; 4) *Safety subcommittee* responsible initially for determining and recommending common procedures for adverse event and serious adverse event responses and reporting procedures; and 5) *Publications subcommittee* responsible initially for considering and recommending collaborative publications of the CHART network.

Consortium communications are coordinated by the RCU. The RCU established and maintains a password-protected communications website to facilitate communications and document sharing across the CHART projects. The RCU also coordinates the biannual in-person meetings and the monthly teleconference meetings of the CHART SC as well as the meetings of the various subcommittees.

### CHART DSMB

The CHART DSMB, constituted by the NHLBI Director, consists of six members who meet twice a year and are charged with advising the NHLBI regarding study design and statistical issues, data control, participant safety and adverse events, and the operational aspects of the trials. The CHART DSMB follows NHLBI DSMB guidelines [6]. Following an open session that includes project PIs and other study staff as required, DSMB members along with the NHLBI executive secretary and biostatisticians from each project meet in closed session to review blinded materials, then the biostatisticians are excused and the DSMB meets in executive session to discuss issues and generate recommendations. The CHART DSMB convened initially on 5 to 6 April 2011 to review and approve the six protocols formally in the CHART network.

### Common baseline and inclusion/exclusion criteria

To facilitate pooled data analyses, the CHART SC considered three tiers of measures:

Tier 1: Variables that all CHART projects will measure using the same procedures;

Tier 2: Variables that each CHART project has the option to measure, but those opting to measure these variables will do so using the same procedures;

Tier 3: Variables unique to each CHART project.

Common baseline inclusion/exclusion, patient-reported, and medical record Tier 1 measures approved by the CHART SC are shown in Table 2. In the process of developing these common baseline measures, the CHART SC and the Design and Analysis subcommittee balanced the numerous possible predictor or moderator hypotheses that could be considered with the response burden of completing these measures, especially for often acutely ill hospitalized patients. Additionally, a core aim of the CHART projects is to produce results that can be implemented easily in hospital systems, and algorithms involving the assessment of multiple moderators to personalize treatment introduces complexity that may hinder implementation. Therefore, only 13 baseline patient reported variables (three for inclusion/exclusion purposes) and 10 medical record variables are included in Tier 1. For Tier 2, those projects measuring nicotine dependence, alcohol use, depression, or quality of life agreed to use the Heavy Smoking Index [7], Audit-C [8], PHQ-2 [9], and the EQ-5D-5 L [10], respectively. Projects can use longer forms of these measures (for example, FTND instead of the Heavy Smoking Index, PHQ-9 instead of PHQ-2), but the shorter forms constitute the shared Tier 2 dataset.

**Table 2:** Tier 1 common baseline measures of the Consortium of Hospitals Advancing Research on Tobacco (CHART) network

**Hospitalization medical record data**	
1	Length of stay (hours, calculated from admission and discharge date/time)
2	Height (cm or in)
3	Weight (kg or lbs)
4	Insurance (public, private, none)
5	Primary and secondary discharge diagnoses (ICD-9)
6	Diagnostic related groups
7	Procedure codes
8	Admission through emergency room (yes, no)
9	Hospital service at admission
10	Discharge plan (home, skilled nursing, rehab)
Eligibility criteria measures	
1	Patient age (via medical record or patient report)
2	Did you smoke a cigarette (even one puff) in the past 30 days? (yes, no)
3	What is your plan about smoking after you leave the hospital? (I plan to quit when I leave the hospital, I plan to try to quit when I leave the hospital, I don’t know if I’m going to quit, I do not plan to quit)
Baseline Patient Report	
1	Are you of Hispanic, Latino, or Spanish origin (yes, no)
2	What is your race? (standard NIH response options)
3	What is your sex? (male, female, other)
4	What is the highest level of education that you havecompleted? (< high school, high school, general equivalency exam, some college, 4 year college graduate or higher)
5	What is your marital status? (married/domestic partner, separated, divorced, widowed, never married)
6	In the past 30 days, on how many days did you smoke? (1 to 30)
7	On the days you smoked, how many cigarettes on average did you smoke?
8	Did you use any other tobacco product besides cigarettes in the past 30 days?
9	How confident are you that you will be able to quit/stay quit once you are discharged from the hospital (5 point scale from “not at all confident” to “very confident”)
10	Does anyone in your household smoke (other than you)? (yes/no/live alone)

ER, emergency room; HS, high school; GED, general equivalency exam.

The CHART Cost-Effectiveness subcommittee developed a cost-effectiveness analysis plan that allows the CHART network to pool data on the cost of intervention delivery, health care utilization of participants during the 1 year post-discharge, and estimates of quality-adjusted life years in those projects administering health utility measures. All projects are estimating intervention costs for cost-effectiveness analyses from a health system (hospital, insurer) perspective using Tier 1 measures and procedures for estimating total cost, cost per patient, and cost per quit for each intervention arm, and incremental cost-effectiveness ratios between intervention arms. Health care utilization and/or expenditures, primarily focused on inpatient, outpatient, and emergency department encounters, will be assessed by all projects via participant report at 6 and 12 months, and costs for utilization will be estimated from diagnostic-related, group-specific Medicare cost weights and other national cost estimates. Projects involving closed systems (for example, HMOs, VA) will also be able to extract actual health care utilization and costs from medical and claims records, and these data will be used for comparison and for cost estimate and utilization adjustments as appropriate.

For inclusion/exclusion criteria, the CHART SC agreed that all projects will define a smoker as any patient who reports having smoked cigarettes, even one puff, in the 30 days prior to hospital admission. This criterion is consistent with most cessation studies of hospitalized smokers [1] and, although it may include some light or intermittent smokers, this criterion insures the inclusion of regular smokers who have not smoked recently due to illness.

For exclusion criteria, the CHART projects are designed to be as inclusive as possible, excluding primarily those patients who are not able to grant informed consent and complete the screening questions due to illness severity, cognitive impairment, and/or illness-related communication difficulties. Some of the CHART projects exclude specific hospital services or units (for example, psychiatric, neurological, and intensive care) while others do not. The interventions provided by some of the projects necessitated additional exclusion criteria (for example, minimal smoking rates for nicotine replacement therapy (NRT) , and access to the internet post-discharge to access the web-based intervention), and some projects are designed to include only those interested in quitting or staying quit while others include all smokers. To facilitate combining data across projects with different exclusion criteria, the CHART projects will assess these exclusion criteria whether or not the individual project excludes on that basis, thus allowing the CHART group to analyze pooled data based on the most stringent exclusion criteria across all projects, and to perform sensitivity analyses on the effects of including versus excluding patient groups excluded by some projects.

To facilitate combining common measures across projects, the RCU will develop variable and value labels to be used by all projects for the Tier 1 and 2 variables. Local projects may use any local data system that will allow production of data sharing files using one of the following formats: SAS, SPSS, or STATA. To ensure consistency, the RCU staff will review plans for collecting common measures, data entry procedures, and data storage formats for each project before the initiation of data collection. The RCU also will create a secure data transfer website for common measures that will allow authorized users to post data and retrieve files.

### Common outcome measures

The CHART projects proposed a range of cessation outcomes, including continuous, prolonged, and 7- and 30-day point prevalence abstinence at 6 and 12 months as well as survival analyses. For a common primary outcome, the CHART SC agreed to 30-day point prevalence at 6 months following hospital discharge. The 6-month follow-up is expected to have less missing data than 12 months, conforms to the minimal recommended follow-up for smoking cessation trials [11,12], and is consistent with recent data showing that the relapse curve of smokers hospitalized for acute coronary syndrome becomes asymptotic within 6 months of admission [13]. The 30-day point prevalence criterion was selected as the primary cessation outcome in part for consistency with the inclusion criteria (that is, participants are defined as a smoker if they smoked within the past 30 days, whether in the 30 days prior to hospitalization or within 30 days of the follow-up assessment), and other studies of hospitalized smokers (for example, PREMIER [14]) also have defined smoking cessation as not smoking within 30 days of the 6 month follow-up.

Although prolonged abstinence is recommended as the primary outcome for smoking cessation studies [11,12], its definition and meaning in the context of interventions for hospitalized smokers is complex. One rationale for prolonged abstinence is the improved confidence that cessation is the result of the intervention given their temporal proximity. The duration of the various CHART interventions, however, range from 4 to 26 weeks post-discharge, making it difficult to define a common period during which most would attempt to quit, or a common grace period in which slips immediately following a quit attempt might occur. Those ready to quit are likely to attempt to continue the abstinence initiated during hospitalization and may slip for a short period after discharge but otherwise remain abstinent. For this group, prolonged abstinence with a 2- to 4-week grace period post-discharge is reasonable. Some of the CHART studies, however, include all smokers including those with little to no interest in quitting after discharge, some of whom may later quit as a result of the continued intervention provided, but these would be defined as failures based on the prolonged abstinence definition above. Given the varying effects of these different interventions on heterogeneous subgroups of hospitalized smokers, the CHART SC decided that 30-day point prevalence, not prolonged abstinence, should be the common primary outcome. Via pooled analyses, the CHART network will be able to compare continuous, prolonged, 30- and 7-day point prevalence abstinence across a large and diverse sample of hospitalized patients and be able to better describe the relationship between these various definitions of cessation outcome.

The CHART SC considered a range of abstinence validation procedures for the 7-day point prevalence outcome at 6 months including mailed and in-person saliva sample collection with cotinine determined via standard laboratory procedures or via test strips, in-person expired carbon monoxide for those reporting continued NRT use, and even proxy report when biochemical validations could not be obtained. Regardless of procedure, the CHART SC remained concerned that the rates of validation completion would be less than optimal and result in a substantial percentage of abstinent participants misclassified as smokers due to failure to obtain biochemical validation samples. A previous study of hospitalized smokers illustrates the competing concerns of missing biochemical samples in those reporting abstinence and misclassifying smokers as abstinent if biochemical verification is not obtained. Hennrikus and colleagues [15] were able to obtain saliva samples from 71.7% of hospitalized smokers who reported abstinence at 12 months, and 19.9% of these samples disconfirmed self-reported abstinence. Across cessation studies, self-report tends to overestimate abstinence compared to biochemically determined abstinence, with an average sensitivity of 86% compared to saliva cotinine and considerable variability in sensitivity between studies [16]. The Society for Research on Nicotine and Tobacco biochemical verification guidelines indicate that “in large-population, low-intensity intervention trials, biochemical validation is neither feasible nor necessary”, but also indicate that medical patients with smoking-related diseases represent a special population in which biochemical verification is recommended [17].

To address the biochemical validation question, the CHART SC decided to rely on self-reported abstinence as the primary measure and conduct a substudy in which a sample of abstinent-reporting participants will receive intensive efforts to obtain biochemical validation at 6 months. The goal of these intensive efforts (for example, considerable incentives, additional staff effort) is to obtain the highest possible rate of completed biochemical validations on this sample of those reporting abstinence. These data will be used by the CHART network to estimate the validated abstinence rates across the full sample and by intervention and control conditions, and provide the smoking cessation research community with data on the sensitivity of self-report in this large sample of hospitalized smokers.

Participating projects will identify participants reporting 7-day point-prevalence abstinence at 6 months. If not on nicotine replacement, these participants will receive materials for collecting a salivary sample to return via mail. Up to five phone or mail reminders will be performed to obtain the sample, and an in-home visit will be offered to those within an hours commute of study staff if the sample is not obtained by the fourth phone or mail contact. These samples will be frozen and batched shipped to Salimetrics (State College, PA, USA) for analysis using enzyme immunoassay. A cut-point of 15 ng/ml will be used to differentiate smokers and non-smokers but sensitivity analyses will be performed with lower recommended cut-points as well. Based on initial power analyses, 442 participants will provide 80% power to detect a 15% difference in misreporting between intervention and control groups, and over 95% power to estimate the overall misreporting rate to within 5%. For the smaller CHART studies, the sample to contribute to this substudy will approximate the full sample of 7-day point prevalence abstinent participants at 6 months, so many of these studies plan on conducting these procedures for all 7-day abstinent participants.

### CHART intervention and comparison conditions

As noted previously, the CHART projects vary considerably in the components, dose, timing, and mode of delivery of the smoking cessation interventions being evaluated, and it was not the intent of the initiative that the interventions should be harmonized. Although the active intervention conditions are not harmonized across projects, the CHART network agreed that the comparison condition should be usual care across all projects. In addition to improving the likelihood of finding a difference between the active and control conditions, requiring usual care as the comparison condition for all studies provides the opportunity to pool data from the usual care conditions across hospitals to document and study the effects of usual care smoking cessation delivered by a variety of hospital systems. The CHART network anticipates that usual care will differ substantially, not only between hospitals but also over time within hospitals, particularly as a result of new Joint Commission requirements on the provision of smoking cessation services in hospital settings. Although this heterogeneity between and within usual care will limit efforts for pooled comparisons of usual care to the various interventions, it will provide a detailed documentation of usual care for smoking cessation in a range of hospital settings and allow for analyses of those components of usual care that may be associated with better outcomes.

## Discussion and conclusion

The CHART network is a consortium of an RCU, six U01s and an associated RC1 project whose primary aim is to study the effectiveness and cost-effectiveness of smoking cessation interventions initiated during hospitalization and continued post-discharge. Over the 4-year project, the NIH (NHLBI, NCI, NIDA, and OBSSR) plans to invest over $20 million in this network of studies. The network will study cessation interventions in nearly 20 hospitals that represent a diverse array of patient demographics and health care resources. The CHART network has developed a set of common outcomes, baseline measures, and inclusion/exclusion criteria to facilitate pooled data analysis of approximately 10,000 patients expected to participate in the studies.

CHART and other similar consortia represent a middle ground between independent studies and multisite standard protocols. The CHART projects are technically independent of one another and have “site rights” to decide what is best for their study. The study investigators, however, have also agreed to work together to build consensus on common measures, inclusion criteria, and other aspects of study protocol to facilitate data sharing and pooling. Limitations of this approach include the inability to directly compare conditions across projects since each project has different intervention and comparison conditions. This necessitates the coding of intervention components and associating these components with outcomes after controlling for project-specific and participant-specific factors to estimate the effects of these various treatment components. The strength of this type of consortia, however, is the ability to plan for harmonizing and pooling common data elements, providing a shared dataset of sufficient size to evaluate possible moderators and mediators of treatment effect that no independent study alone could perform.

The CHART network is also an example of designing for dissemination. The projects are evaluating interventions that can be easily implemented in hospital settings, and dissemination and implementation issues have been an important consideration in all CHART network decisions. When completed, the CHART studies should provide hospital systems with a number of effective smoking cessation interventions, at least one of which can serve as the basis for a smoking cessation intervention that fits with the resources and infrastructure of their hospital system. Pooled data on the relationship of outcomes to various intervention components, both for the intervention and usual care conditions, should provide additional guidance on the relative importance of various intervention components for maximizing cessation outcomes. These pooled analyses should also give hospital systems with limited resources guidance on which patient groups are more likely to benefit from these interventions and possible stepped interventions based on intervention intensity needed to support cessation. The knowledge gained from the CHART initiative should have a significant impact on the delivery of smoking cessation interventions in hospital settings.

## Abbreviations

AMI, acute myocardial infarction; CHART, Consortium of Hospitals Advancing Research on Tobacco; DSMB, Data and Safety Monitoring Board; NCI, National Cancer Institute; NHLBI, National Heart, Lung, and Blood Institute; NIDA, National Institute on Drug Abuse; NIH, National Institute of Health; NRT, nicotine replacement therapy; OBSSR, Office of Behavioral and Social Science Research; PI, Principal Investigator; QALY, quality-adjusted life years; RCU, research coordinating unit; RFA, request for application; SC, Steering Committee.

## Competing interests

The authors declare that they have no competing interests.

## Authors’ contributions

WR is the NHLBI project director and led the writing of this manuscript. VS is the PI for the RCU responsible for the coordination across the CHART projects. SZ is the chair of the CHART Design and Analysis Committee and is responsible for leading the common measures and design effort across projects. GM and DG are project scientists from NCI and NIDA, respectively, and have contributed to this initiative since its inception. All authors read and approved the final manuscript.
